# Transforming Growth Factor-β: Activation by Neuraminidase and Role in Highly Pathogenic H5N1 Influenza Pathogenesis

**DOI:** 10.1371/journal.ppat.1001136

**Published:** 2010-10-07

**Authors:** Christina M. Carlson, Elizabeth A. Turpin, Lindsey A. Moser, Kevin B. O'Brien, Troy D. Cline, Jeremy C. Jones, Terrence M. Tumpey, Jacqueline M. Katz, Laura A. Kelley, Jack Gauldie, Stacey Schultz-Cherry

**Affiliations:** 1 Department of Medical Microbiology and Immunology, University of Wisconsin, Madison, Wisconsin, United States of America; 2 Pfizer Inc., Department of Viral Vaccines, Research Triangle Park, North Carolina, United States of America; 3 Department of Infectious Disease, St. Jude Children's Research Hospital, Memphis, Tennessee, United States of America; 4 Influenza Division, National Center for Immunization and Respiratory Diseases, Centers for Disease Control and Prevention, Atlanta, Georgia, United States of America; 5 Biosciences Research Laboratory, USDA Agricultural Research Station, Fargo, North Dakota, United States of America; 6 Department of Pathology and Molecular Medicine, McMaster University, Hamilton, Ontario, Canada; Erasmus Medical Center, Netherlands

## Abstract

Transforming growth factor-beta (TGF-β), a multifunctional cytokine regulating several immunologic processes, is expressed by virtually all cells as a biologically inactive molecule termed latent TGF-β (LTGF-β). We have previously shown that TGF-β activity increases during influenza virus infection in mice and suggested that the neuraminidase (NA) protein mediates this activation. In the current study, we determined the mechanism of activation of LTGF-β by NA from the influenza virus A/Gray Teal/Australia/2/1979 by mobility shift and enzyme inhibition assays. We also investigated whether exogenous TGF-β administered via a replication-deficient adenovirus vector provides protection from H5N1 influenza pathogenesis and whether depletion of TGF-β during virus infection increases morbidity in mice. We found that both the influenza and bacterial NA activate LTGF-β by removing sialic acid motifs from LTGF-β, each NA being specific for the sialic acid linkages cleaved. Further, NA likely activates LTGF-β primarily via its enzymatic activity, but proteases might also play a role in this process. Several influenza A virus subtypes (H1N1, H1N2, H3N2, H5N9, H6N1, and H7N3) except the highly pathogenic H5N1 strains activated LTGF-β *in vitro* and i*n vivo*. Addition of exogenous TGF-β to H5N1 influenza virus–infected mice delayed mortality and reduced viral titers whereas neutralization of TGF-β during H5N1 and pandemic 2009 H1N1 infection increased morbidity. Together, these data show that microbe-associated NAs can directly activate LTGF-β and that TGF-β plays a pivotal role protecting the host from influenza pathogenesis.

## Author Summary

Transforming growth factor-beta (TGF-β) is a multifunctional protein that serves as a global regulator of immunity by controlling the initiation and resolution of inflammatory responses. A pathogen that can regulate TGF-β activation could promote an immune-privileged state for itself within its host. Indeed, multiple parasitic, bacterial, and fungal pathogens successfully evade immune responses by regulating TGF-β. We demonstrate that the neuraminidase proteins from influenza A viruses and *Clostridium perfringens* convert biologically inactive TGF-β to its active form. Importantly, modulation of TGF-β activity during influenza infection affects viral titers and disease outcome in mice, suggesting that TGF-β plays an important role in influenza pathogenesis, particularly in protecting the host during infection. These studies suggest that neuraminidases from diverse microbes may be able to directly regulate TGF-β, which may in turn play an important role in disease.

## Introduction

Transforming growth factor-β1 (TGF-β) is the prototypic member of a family of multifunctional cytokines that modulate diverse cellular, developmental, and immunological processes (reviewed in [1]–[3]). TGF-β is secreted by virtually all cells as a biologically inactive molecule termed latent TGF-β (LTGF-β) [4], [5]. The latent complex consists of an N-terminal latency-associated peptide (LAP) and the mature TGF-β domain. LAP and TGF-β are products of a single gene, which after posttranslational modifications such as glycosylation and phosphorylation and cleavage by furin remain noncovalently associated, forming the small latent complex [6]. The small latent complex is secreted by cells as an inactive complex, and in some cases is linked by a disulfide bond to the latent TGF-β-binding protein to form the large latent complex.

The non-covalent association of LAP with the mature domain is critical for latency. The molecular mechanism by which LAP confers latency to mature TGF-β is largely unknown. However, recent studies suggest that amino acids 50–85, several of which are glycosylated and contain terminal sialic acid residues, are critical for proper formation and function of the LTGF-β complex [7]. Mutations in this region reduce the binding of LAP to the mature domain and significantly impair the ability of LAP to confer latency to mature TGF-β [8]. Agents that activate the latent complex can disrupt the association of LAP with the mature domain either by proteolysis or denaturing the LAP or by altering its folding [6]. Given the abundance of LTGF-β and the prevalence of high-affinity receptors on most cell types, the activation of LTGF-β is recognized as a crucial step in TGF-β function (reviewed in [9], [10]).

Chaotropic agents, heat, reactive oxygen species [11], [12], and extreme pH [13], [14] can activate LTGF-β. *In vitro* studies have identified proteases, which degrade the LAP (reviewed in [15]), and molecules such as thrombospondin-1, which alter the conformation of the LAP [16], [17], [18], [19], [20], as putative physiological TGF-β activators. Less is known about activation *in vivo*, although integrins appear to be the primary LTGF-β activators in the lung [10], [21], [22].

Little is known about the direct activation of LTGF-β by microbes. Several parasites such as *Trypanosoma cruzi*, *Leishmania* spp., and *Plasmodium* spp. and the bacteria *Mycobacterium tuberculosis* activate LTGF-β through proteolysis, using either host-derived plasmin or microbe-encoded proteases [23]–[26]. In a previous study, we have shown that influenza viruses activate TGF-β *in vitro* and *in vivo* [27]. Antibodies to the viral neuraminidase (NA) protein inhibited viral-induced LTGF-β activation, suggesting that NA plays a role in LTGF-β activation, but the precise mechanism of activation remains to be identified and the role of TGF-β in influenza disease is unknown. In this study, we determined the mechanism of activation of rLTGF-β by viral and bacterial NA. Since NA is essential for viral replication, we tested a panel of influenza virus subtypes (including two 2009 H1N1 pandemic strains) for their ability to activate LTGF-β *in vitro*. For strains that failed to activate LTGF-β, we used reverse genetic studies to determine whether these viruses had deficient NA activity. We also investigated whether exogenous TGF-β provides protection from H5N1 influenza pathogenesis and whether depletion of TGF-β during virus infection increases morbidity in mice.

## Results

### Purified NA activates LTGF-β

**Figure 1:**
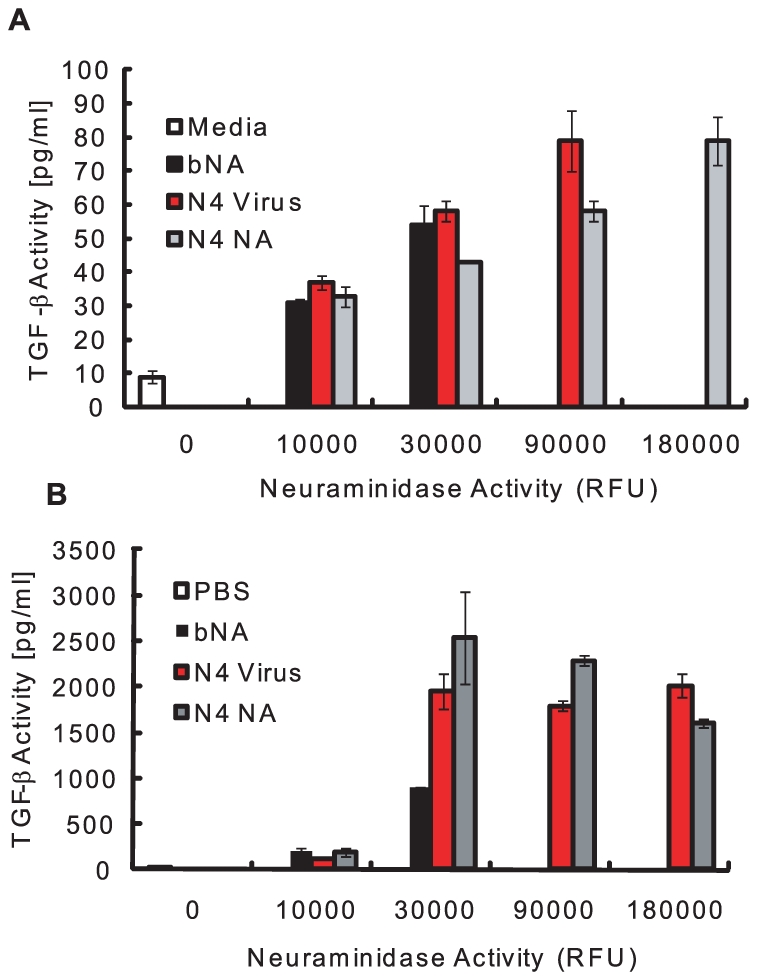
NA activates LTGF-β. rLTGF-β (10 ng/ml) was incubated alone (white bar) or with increasing amounts of enzymatically equivalent amounts of bacterial NA (bNA, black bar), N4 virus (red bar), or purified Gray Teal NA (gray bar) for 1 h at 37°C and TGF-β activity (pg/ml) measured by the PAI/L assay (A) or ELISA (B). Error bars represent standard error of the mean.

To begin defining the mechanism of NA-mediated activation of LTGF-β, we first asked if NA purified from the virion was sufficient for activation. Thus, recombinant LTGF-β (rLTGF-β) was incubated with buffer alone, purified A/Gray Teal/Australia/2/1979 virus (N4 virus), purified Gray Teal NA (N4 NA, BEI Resources, Manassas, VA), or low-protease-content NA purified from *Clostridium perfringens* (Roche), which was used as a non-viral NA control (bNA). All the samples were standardized to equivalent NA enzymatic activity and rLTGF-β activation was monitored by two different assays; the PAI/L bioassay, which monitors the activation of a TGF-β-specific reporter construct expressed in a stable cell line, or a sandwich ELISA specifically recognizing an epitope on the active TGF-β protein. All of the samples activated rLTGF-β in both assays in a dose-dependent manner. In the PAI/L bioassay, the concentration of active TGF-β increased with increasing amounts of NA (Fig. 1A). However, at the highest concentration of N4 virus (180,000 RFU) there was no TGF-β activity and the cells appeared dead. In the ELISA, both the N4 virus and purified NAs had low, but detectable levels of TGF-β activity at the lowest dose tested (10,000 RFU), that increased at 30,000 RFU, and then remained steady at the higher NA concentrations (Fig. 1B). Overall, these studies demonstrate that both influenza viral and a bacterial NA can activate LTGF-β.

### NA removes sialic acids on the LAP

**Figure 2:**
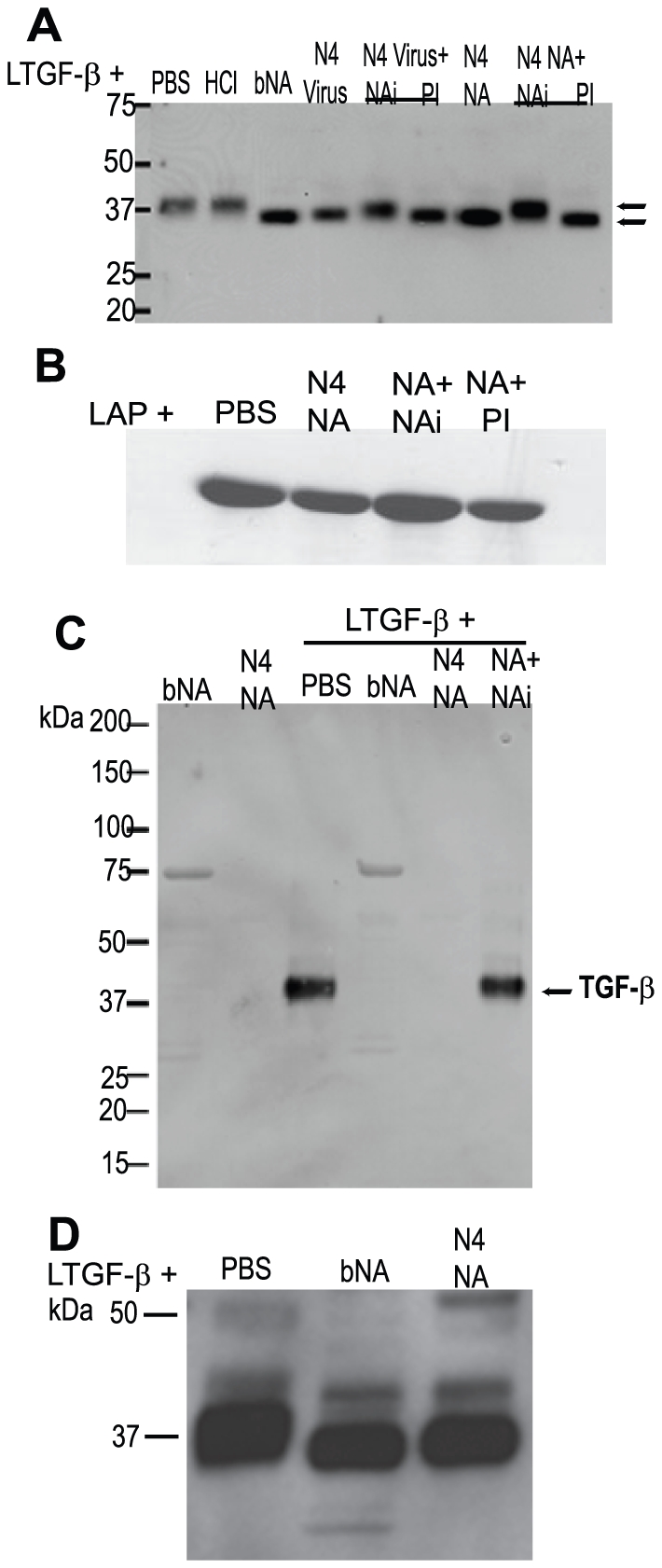
NA induces a shift in the size of LAP and removes sialic acids from the LAP. rLTGF-β (0.4 µg) was incubated with PBS, HCl (pH 2), bacterial NA (bNA), purified Gray Teal virus (GT Virus, 2 µg), or purified Gray Teal NA (GT NA, 0.5 µg) in the presence or absence of 1 µM oseltamivir carboxylate (NAi) or 1× protease inhibitor cocktail (PI) for 1 h at 37°C. Proteins were separated by SDS-PAGE under reducing conditions, transferred to nitrocellulose, and probed with anti-LAP antibody (A and D) or DIG-labeled MAA lectin (C) by Western blot analysis. (B) Insect cell–derived rLAP (0.5 µg) was incubated as described above and probed with anti-LAP antibody by Western blot analysis. bNA and GT NA alone were run as controls for (C).

To determine if NA-mediated activation involved removal of the sialic acid motifs on the LAP, rLTGF-β was incubated with PBS, bNA, N4 virus, or purified N4 NA, and the size of the LAP was determined by Western blot. HCl was used as a control for non-enzymatic-mediated activation of rLTGF-β. The rLTGF-β incubated with bNA, N4 virus, and N4 NA showed a slight shift in mobility of the LAP as compared to that incubated with PBS or HCl (Fig. 2A). There was no significant difference in the mobility between the bNA, N4 virus, and N4 NA. This shift in mobility was not evident when N4 NA was incubated with rLTGF-β purified from insect cells (Fig. 2B). The rLTGF-β produced by insect cells is unsialylated, as insect cells have no detectable sialyltransferase activity [28]. Thus, the lack of size change upon incubation with N4 NA suggests that the increased mobility of LTGF-β treated with N4 virus, bNA, or N4 NA is due to removal of sialic acid moieties. To confirm this, rLTGF-β was incubated with PBS, bNA, or N4 NA, proteins separated on a reducing SDS-PAGE, and sialic acid expression monitored by Western blot analysis using digoxigenin (DIG)–labeled lectins *Maackia amurensis* agglutinin (MAA; recognizes α2-3 sialic acid linkages, Fig. 2C) or *Sambucus nigra* agglutinin (SNA; recognizes α2-6 sialic acid linkages, Fig. S1). Blots were also probed with anti-LAP to confirm that the protein analyzed was the LAP (Fig. 2D). rLTGF-β incubated with PBS was detected by both SNA and MAA, suggesting the presence of both α2-6 and α2-3–linked sialic acids on the LAP (Fig. 2C and Fig. S1). In contrast, MAA and SNA failed to recognize bNA-treated rLTGF-β. Similarly, N4 NA–treated rLTGF-β was not recognized by MAA (Fig. 2C), but was detected by SNA (Fig. S1). However, this does not imply that the N4 NA fails to cleave α2-6 linkages. Hence, the mobility shift observed when rLTGF-β was incubated with NA is likely because of the loss of LAP-associated α2,3-linked sialic acids, but the specific sialic acid linkages removed may depend on the NA, as seen in the case of bNA-treated rLTGF-β.

### NA enzymatic activity is involved in rLTGF-β activation, but proteases may also play a role in this process

**Figure 3:**
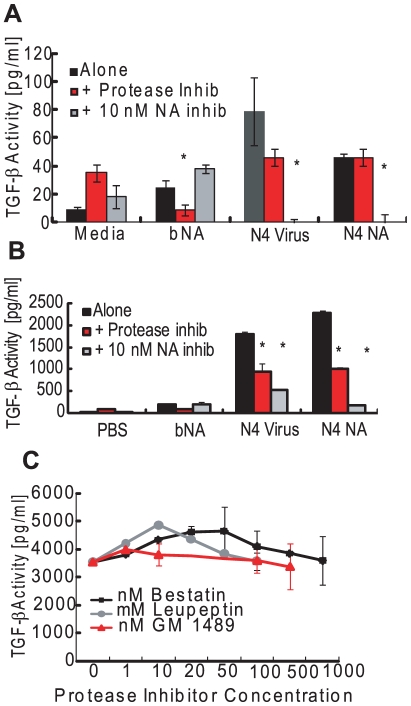
Role for NA activity and proteases in LTGF-β activation by influenza virus. (A) rLTGF-β (10 ng/ml) was incubated alone (black bar) or with bNA (30,000 RFU NA activity), purified N4 virus (90,000 RFU NA activity), or purified N4 NA (90,000 RFU NA activity) in the presence or absence of 1× PI cocktail (red bars) or 10 nM oseltamivir carboxylate (NAi, gray bars) for 1 h at 37°C. TGF-β levels were measured by (A) PAI/L assay or (B) ELISA. (C) rLTGF-β (10 ng/ml) was incubated with purified Tk/WI virus (10^6^ TCID_50_ units/ml) alone or in the presence of increasing concentrations of bestatin (▪, nM), leupeptin (•, µM) or GM1489 (Δ, nM), and TGF-β activity measured by ELISA. Error bars represent standard error of the mean. Asterisk (*) indicates significant inhibition as compared with virus/NA-treated LTGF-β in the absence of inhibitor.

To determine whether the enzymatic activity of NA was required for loss of specific sialic acid motifs and TGF-β activation, N4 virus or NA was pre-incubated with the influenza-specific inhibitor (NAi) oseltamivir carboxylate (10 nM) before incubation with rLTGF-β. Pre-incubation with NAi inhibited the mobility shift in the LAP (Fig. 2A), the loss of sialic acid (Fig. 2C), and TGF-β activation (Fig. 3A and B). N4 virus and NA–induced activation was completely inhibited with 10 nM NAi in the PAI/L assay (Fig. 3A) and to a lesser degree (75–95%) in the ELISA (Fig. 3B). Increasing the concentration of NAi up to 10 µM failed to completely inhibit activation in the ELISA assay (data not shown). Because the NAi is specific for influenza NA, it did not inhibit bNA-induced activation of rLTGF-β (Fig. 3A and 3B).

Proteases are established activators of LTGF-β [15] and can be contaminants of viral preparations or even components of the viral membrane [29], [30]. To examine the role for proteases, the LAP shift and activation assays were performed in the presence of a broad-spectrum protease inhibitor (PI) cocktail. The PI cocktail used in these studies had no effect on sialidase activity of either the virus or NAs and did not inhibit active TGF-β detection in either assay (data not shown). Unlike NAi, pre-incubation with PI had no effect on the LAP mobility shift (Fig. 2A). However, the PI inhibited the N4 virus and NA-induced activation of LTGF-β in the ELISA assay (Fig. 3B) and to some extent in the PAI/L assay (Fig. 3A), although the inhibition was not as much as that seen with NAi treatment. To determine the specific class of proteases causing the inhibition, virus was pretreated with increasing concentrations of individual protease inhibitors within their effective inhibitory ranges and incubated with rLTGF-β, and TGF-β activity was determined by ELISA (Fig. 3C). None of the protease inhibitors blocked rLTGF-β activation by the N4 virus. Further, when incubated with substrate for 1 h, all reagents tested protease-free (negative for trypsin, chymotrypsin, thrombin, plasmin, elastase, subtilisin, papain, cathepsin B, thermolysin, and pepsin) in a fluorescein thiocarbamoyl-casein derivative-based assay kit (data not shown). Even when incubations were extended to 24 h, only few viral stocks were positive for proteases (Fig. S2). Together, these data suggest that NA activates LTGF-β primarily via a mechanism involving enzymatic activity. However, a role for proteases cannot be discounted especially during infection *in vivo*.

### Most influenza strains activate LTGF-β

**Figure 4:**
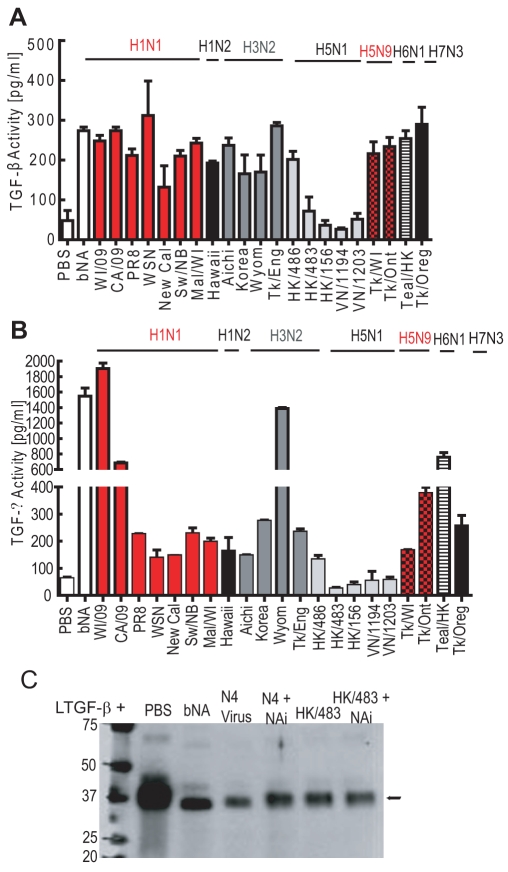
Influenza viruses differentially activate LTGF-β. rLTGF-β (10 ng/ml) was incubated alone (white bar) or with different influenza virus subtypes (90,000 RFU) for 1 h at 37°C and TGF-β activity measured by PAI/L assay (A) or ELISA (B). (C) rLTGF-β (0.4 µg) was incubated with PBS, bNA, N4 Virus (2 µg), or HK/483 virus (HK/483, 2 µg) in the presence or absence of 1 µM oseltamivir carboxylate (NAi) for 1 h at 37°C. Proteins were separated by SDS-PAGE under reducing conditions, transferred to nitrocellulose, and probed with anti-LAP antibody by Western blot analysis. Error bars represent standard error of the mean.

As NA is essential for viral replication, we hypothesized that all influenza strains could activate LTGF-β. rLTGF-β was incubated with a panel of influenza virus subtypes, including two 2009 H1N1 pandemic strains, and several highly pathogenic avian influenza viruses (H5N1 and H5N9), and TGF-β activity was measured by the PAI/L assay (Fig. 4A) or ELISA (Fig. 4B). Although most of the strains activated LTGF-β, the levels of activation differed despite having equivalent NA activity. Surprisingly, several of the H5N1 influenza viruses failed to activate rLTGF-β; only A/Hong Kong/486/1997 (HK/486) consistently activated rLTGF-β (Fig. 4A and 4B). Incubation of rLTGF-β with a representative non-activating H5N1 virus, A/Hong Kong/483/1997 (HK/483), did not cause the expected mobility shift in the LAP (Fig. 4C), suggesting that the NA from viruses that did not activate rLTGF-β may also not cleave sialic acids from the LAP.

### There is no intrinsic defect in the H5N1 viral NA

To determine whether the failure of H5N1 viruses to activate rLTGF-β was due to an intrinsic defect in the H5 NA protein, we first examined rLTGF-β activation by A/Teal/Hong Kong/W312/97 (Teal/HK) H6N1 virus. Teal/HK NA shares 97% sequence nucleotide homology with the H5N1 NA including a 19-amino-acid deletion in the stalk region and is the proposed donor of the NA and the internal genes of the H5N1 viruses [31]. Unlike the H5N1 viruses, Teal/HK virus activated rLTGF-β in both assays (Fig. 4A and 4B) suggesting that the deletion in the NA stalk domain has no effect on TGF-β activation.

**Figure 5:**
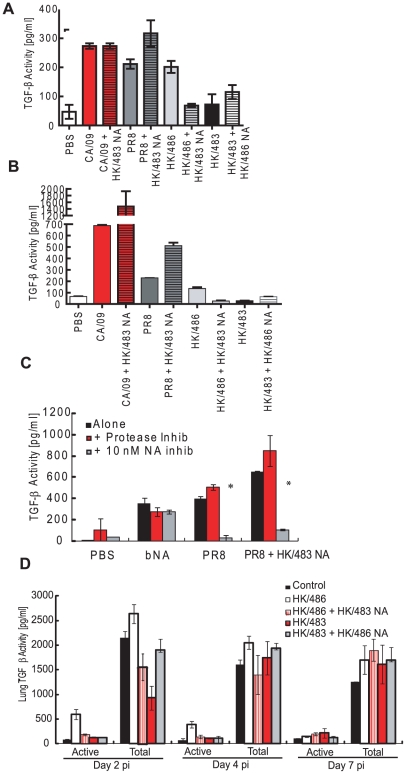
Reassortant viruses differentially activate rLTGF-β. rLTGF-β (10 ng/ml) was incubated alone (white bar), or with equivalent levels (90,000 RFU NA activity) of parental CA/09 (red bars), parental PR8 (dark gray bars) or parental HK/486 (light gray) viruses, or reassortant viruses expressing the HK/483 NA (hatched bars) for 1 h at 37°C and TGF-β activity measured by PAI/L assay (A) or ELISA (B). (C) rLTGF-β (10 ng/ml) was incubated with bNA, parental PR8 virus, or the PR8+HK/483 NA viruses alone (black bars) or in the presence or absence of 1× PI cocktail (red bars) or 10 nM oseltamivir carboxylate (gray bars) for 1 h at 37°C and TGF-β activity measured by ELISA. (D) BALB/c mice (4–6 weeks old) were inoculated i.n. with PBS (control, *n* = 8), or 10^4^ TCID_50_ units of the parental or reassortant viruses (*n* = 10) and lungs collected at 2, 4, and 7 dpi. Total (acid-activated samples) and active TGF-β levels were monitored in the lung homogenates by the mouse TGF-β–specific ELISA. Error bars represent standard error of the mean.

To further assess the H5N1 NA, two H1N1 influenza viruses (A/California/04/09 and A/Puerto Rico/8/34) expressing the HK/483 NA were generated (CA/09+HK/483 NA and PR8+HK/483 NA) and tested for rLTGF-β activation. Both the parental viruses and the reassortant viruses containing the HK/483 NA activated rLTGF-β in the PAI/L (Fig. 5A) and ELISA (Fig. 5B) assays. Further, activation was inhibited by NAi but not the PI cocktail (Fig. 5C), confirming that the HK/483 NA can activate rLTGF-β in a NA-dependent manner.

### H5N1 viral NA fails to activate rLTGF-β on an H5 virus *in vitro* and *in vivo*

To construct an H5N1 virus that activated rLTGF-β, HK/483 virus expressing the HK/486 NA was generated. Unfortunately, this virus was unable to activate rLTGF-β (Fig. 5A and 5B). Further, expressing the HK/483 NA on the HK/486 virus led to reduced activation as compared to the parental HK/486 virus. To evaluate LTGF-β activation *in vivo*, BALB/c mice were intranasally inoculated with PBS (control, *n* = 8) or 10^4^ TCID_50_ units of the different reassortant viruses (*n* = 10) and lungs collected at 2, 4, and 7 days post-infection (dpi). Active or total (determined by acid activation of the sample) levels of TGF-β in the lung homogenates were determined by ELISA (Fig. 5D). Similar to the *in vitro* results, only HK/486 increased TGF-β activity in the lungs of infected mice as compared to PBS-inoculated mice. Levels of active TGF-β were increased >5-fold within 2 dpi, remained elevated at 4 dpi, before returning to control levels at 7 dpi (Fig. 5D). These kinetics were similar to those observed in mice infected with PR8 virus [27] and other highly pathogenic avian influenza viruses [32].

The total TGF-β levels in the lungs remained constant for all the viruses except for a significant decrease (∼60%) in the HK/483 infected mice at 2 dpi (Fig. 5D). This decline was not seen in mice infected with the HK/483+HK/486 NA reassortant virus, which remained at control levels at 2 dpi. These findings suggest that the NA may influence the total levels of TGF-β in the lungs of infected mice through an undefined mechanism.

### Exogenous TGF-β provides partial protection during HK/483 infection

**Figure 6:**
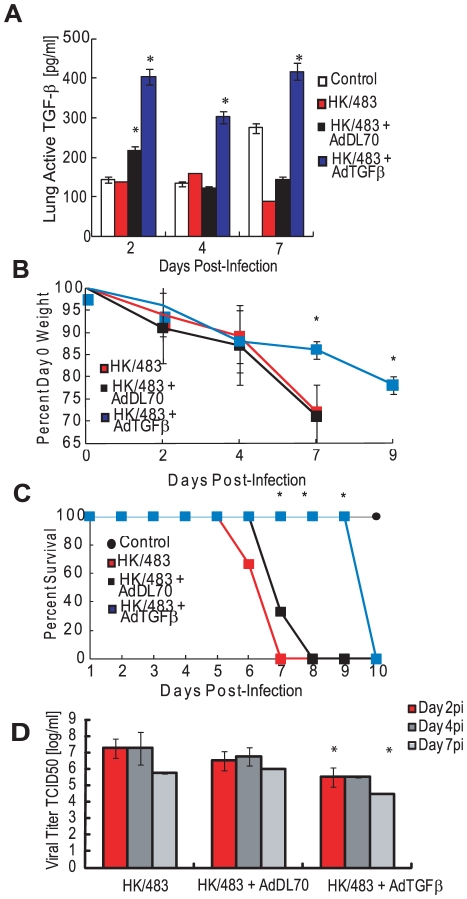
Exogenous TGF-β delays mortality in HK/483-infected mice. BALB/c mice (4–6 weeks old) were inoculated i.n. with PBS (control, *n* = 10), or 10^4^ TCID_50_ units of HK/483 virus and 24 hpi inoculated with 10^8^ PFU/mouse of a TGF-β-expressing adenovirus (AdTGFβ^223/225^) or a control adenovirus vector (AdDL70) (infected groups, *n* = 12). Mice inoculated with AdDL70 or AdTGFβ^223/225^ alone served as controls (*n* = 10). At 2, 4, and 7 days post HK/483 infection (1, 3, and 6 day post-adenovirus administration), lung homogenates were monitored for TGF-β levels by a mouse-specific ELISA (A) and viral titers by TCID_50_ analysis on MDCK cells (D). For titers, red bars, day 2pi; dark gray bars, day 4 pi; and gray bars, day 7 pi. Weights (B) and survival (C) were monitored for 10 dpi. Error bars represent standard error of the mean. Asterisk (*) indicates significant increase in TGF-β levels as compared with other groups (A); difference in weight loss (B) or mortality (C) as compared with HK/483 virus and AdDL70-treated mice; and decrease in viral titers as compared with HK/483 infected mice (D).

Because we were unable to construct an HK/483 H5N1 virus that activates TGF-β *in vivo*, active TGF-β1 was administered to HK/483-infected mice by using a replication-deficient adenovirus vector. Twenty-four hours after HK/483 infection (10^4^ TCID_50_), 10^8^ PFUs of control adenovirus vector (AdDL70, *n* = 12), TGF-β-expressing vector AdTGFβ^223/225^ (*n* = 12), or PBS (*n* = 12) were administered intranasally. Lung TGF-β levels were measured at 2, 4, and 7 dpi. By 2 dpi, TGF-β levels in the lung increased to approximately 450 pg/ml in mice treated with the TGF-β–expressing adenovirus and remained above control levels even at 7 dpi (Fig. 6A). Mice treated with the control virus AdDL70 showed a transient increase in lung TGF-β activity at 2 dpi (100 pg/ml), which returned to control levels by 4 dpi. By 4 dpi with the HK/483 virus, all infected mice lost approximately 15% (*p*<0.01) of their initial body weight, which increased to more than 20% by 7 dpi in the HK/483 and +AdDL70 groups (Fig. 6B), at which time mice either succumbed to infection or were euthanized (Fig. 6C). Mice inoculated with AdTGFβ^223/225^ showed delayed weight loss and prolonged survival. At 7 dpi, weight loss remained at approximately 15% (*p*<0.01), but increased to 25% by 9 dpi (Fig. 6B). This was associated with a significant delay in mortality: AdTGFβ^223/225^- infected mice survived until 10 dpi (*p*<0.05, Fig. 6C). These mice also had significantly lower viral titers than HK/483-infected mice (Fig. 6D). By 2 dpi (1 day post AdTGFβ^223/225^ inoculation), viral titers decreased from approximately 10^7.5^ TCID_50_ to 10^5.5^ TCID_50_ (*p*<0.05) in the HK/483 alone and AdDL70 groups. Similar decreases in titers were observed at 4 and 7 dpi (*p*<0.05) in the HK/483 alone group. However, there was no significant difference in titers between the AdDL70 and AdTGFβ^223/225^ groups at 4 dpi.

Given the increased survival of mice infected with AdTGFβ^223/225^, we tested whether pretreatment with TGF-β afforded additional protection to mice. Mice (*n* = 12) were administered 10^8^ PFUs AdDL70 control or the AdTGFβ^223/225^ virus 48 h before HK/483 infection. Pretreatment with AdTGFβ^223/225^ provided no added protection; all the HK/483-infected mice succumbed to infection by 8 dpi (Fig. S3B). Both the uninfected and HK/483-infected mice pretreated with AdTGFβ^223/225^ lost significantly more weight by 4 dpi than mice in other groups (10% vs. 0%, *p*<0.01, Fig. S3A), suggesting that increased TGF-β activity before H5N1 influenza infection can be detrimental to mice.

### Depletion of TGF-β during HK/486 or pandemic 2009 H1N1 infection increases morbidity

**Figure 7:**
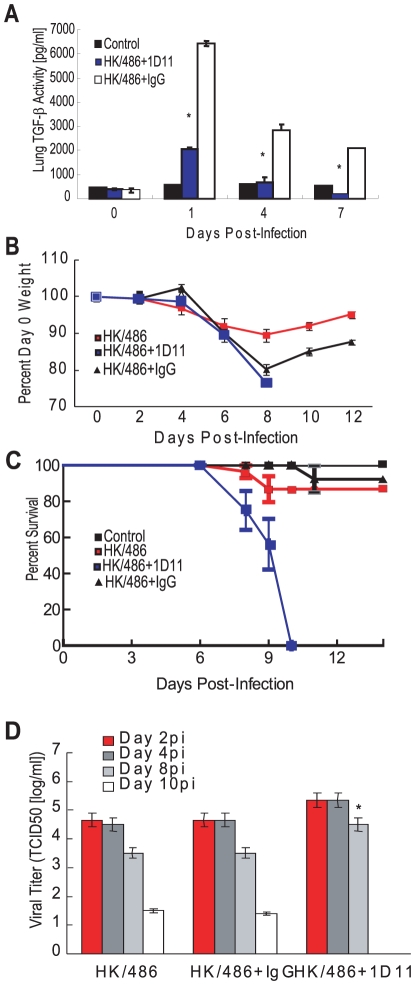
Depletion of TGF-β alters morbidity in HK/486-infected mice. Six hours before infection, BALB/c mice were treated with a TGF-β neutralizing antibody (1D11) or an isotype control antibody (IgG) at a dose of 0.5 mg/mouse and subsequently inoculated with PBS (control) or 10^5^ TCID_50_ units of HK/486 virus (n = 15). Antibodies were readministered every 48 hpi. At 0, 1, 4, and 7 days pi, total TGF-β levels were analyzed in lung homogenates by a mouse TGF-β-specific ELISA (A). Weights (B) and survival (C) were monitored for 14 dpi. On days 2, 4, 8, and 10 pi, lung homogenates were monitored viral titers by TCID_50_ analysis on MDCK cells (D). Error bars represent standard error of the mean. Asterisk (*) indicates significant decrease in TGF-β levels as compared with infected group treated with IgG (A), and increase in viral titers as compared with HK/486-infected mice with and without IgG treatment (D).

We then examined the effect of removing TGF-β during HK/486 infection by depleting TGF-β using a pan-TGF-β neutralizing antibody. Briefly, 1D11 antibody or isotype-control IgG was administered and total TGF-β levels in the lungs were monitored by ELISA (Fig. 7A). Total TGF-β levels in HK/486-infected mice were significantly (3 times) lower (*p* = 0.0003) by 24 hpi than in the HK/486-infected mice receiving isotype IgG. Two doses of the neutralizing antibody decreased TGF-β levels to control levels; by 7 dpi, levels were significantly (3 times) lower (*p* = 0.04) than control levels. By 8 dpi, all HK/486-infected mice (10^5^ TCID_50_, *n* = 15) lost approximately 20% of their starting weight whereas HK/486-alone mice lost only 10% (Fig. 7B, *p*<0.01). By 9 dpi, 40% of mice in the TGF-β–depleted group succumbed to infection, and all died by 10 dpi (Fig. 7C), whereas those in the HK/486 and isotype IgG groups began to recover and gain weight. The increased mortality in the TGF-β–depleted group was not associated with a significant increase in viral replication. HK/486-infected mice with and without isotype IgG had peak lung titers of approximately 10^4.6^ TCID_50_ by 2 dpi, which decreased to 10^1.5^ TCID_50_ by 10 dpi (Fig. 7D). In contrast, the TGF-β–depleted group had a slight, although not significant, increase in viral titers at 2 and 4 dpi. At 8 dpi, 1D11-treated mice had a 15-fold increase in viral titers over HK/486 and isotype IgG–treated mice (*p*<0.02). No virus was detected in control tissues or outside the lungs of infected mice.

**Figure 8:**
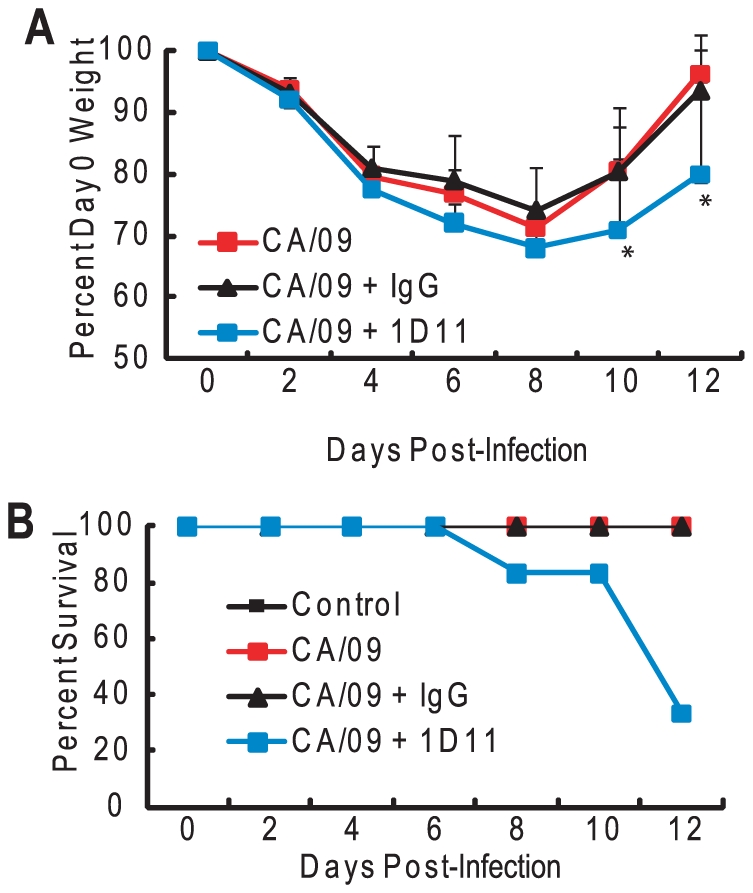
Depletion of TGF-β alters morbidity in HK/486-infected mice. Forty-eight hours before infection, BALB/c mice were treated with a TGF-β neutralizing antibody (1D11) or an isotype control antibody (IgG) at a dose of 0.5 mg/mouse and subsequently inoculated with PBS (control) or 10^5^ TCID_50_ units of CA/09 virus (n = 6). Antibodies were re-administered every 48 hpi. Mice were monitored daily for weight loss (A) and mortality (B). Error bars represent standard error of the mean. Asterisk (*) indicates significant weight loss as compared to HK/486 infected mice.

To determine if these findings were specific to the highly pathogenic H5N1 influenza viruses, mice (*n* = 6) were pre-treated with PBS, the 1D11 antibody or isotype-control IgG as described, infected with A/California/04/09 (CA/09, 10^5^ TCID_50_), and monitored for morbidity. The CA/09-infected mice treated with PBS or receiving the isotype IgG lost approximately 20–25% of their starting weight by 6 to 8 dpi before returning to day 0 weights by 12 dpi (Fig. 8A). Clinically the mice had ruffled fur and were shivering. The 1D11-treated mice followed a similar pattern but lost significantly more weight by 6 dpi (*p* = 0.047) and had a delayed recovery with significantly more weight loss still evident at 12 dpi (*p* = 0.029). The 1D11-treated mice had more significant clinical signs of infection including rear-leg paralysis and had 20% mortality by 8 dpi reaching 67% by 12 dpi (Fig. 8B). Taken together, the data suggest that TGF-β is modulated by the virus, and this modulation during infection may be important in disease outcome.

## Discussion

These studies establish NA as a direct activator of LTGF-β and demonstrate a role for TGF-β in protection against influenza virus pathogenesis. We have previously shown that purified influenza virus activates TGF-β [27] and that antibodies to the viral NA but not the HA inhibit viral-mediated LTGF-β activation. In this study, we demonstrate that purified NA alone can convert the biologically latent form of TGF-β to its active form and that TGF-β plays an important role in protection against influenza virus pathogenesis. Activation of LTGF-β by viral NA involves removal of sialic acid moieties to release the active TGF-β molecule from the latent complex or expose other residues for cell surface interactions. To our knowledge, these are the first studies demonstrating that microbe-associated sialidases can directly activate LTGF-β.

Our study also shows that NA-mediated LTGF-β activation is not specific to influenza virus. As the topology of the NA catalytic domain is well conserved and the active sites share many structural features [33], NAs from diverse pathogens may activate LTGF-β. In our study, *Clostridium perfringens*–derived bNA also activated LTGF-β, which is consistent with previous studies showing LTGF-β activation by bNA, although sialidase activity was not explicitly identified as the means of activation [34], [35]. Paramyxoviruses, which also have a functional NA protein, directly activate LTGF-β (unpublished data). A study by Zou and Sun demonstrated that LTGF-β2 and LTGF-β3 were also activated by NA [36], suggesting that NA may be a biological activator of numerous types of LTGF-β.

Despite the functional conservation among NAs, some highly pathogenic avian (HPAI) H5N1 influenza viruses failed to activate LTGF-β *in vitro* and *in vivo* [32], [37]. The NA from these viruses has a 19-amino-acid deletion in the stalk [31], [38] that could contribute to the decreased ability to activate LTGF-β. To test this possibility, we assessed the activation of rLTGF-β by the Teal/HK H6N1 virus. Hoffmann et al., proposed that this virus may have donated the NA gene to the H5N1 viruses given the high degree of nucleotide homology [31]. In spite of the stalk deletion, Teal/HK activated rLTGF-β unlike the H5N1 viruses. Further, expressing the HK/483 NA on either PR8 or CA/09 virus had no effect on rLTGF-β activation suggesting that there is no intrinsic defect in the NA.

However, expressing the HK/483 NA on the H5 HK/486 virus led to an inability to activate LTGF-β *in vitro* and *in vivo*. In addition the HK/486 NA failed to rescue the activation phenotype with the HK/483 virus. A recent study by Matsuoka et al demonstrated that short-stalk NAs from the H5N1 viruses are more virulent in mice and chickens. Intriguingly, the NA-mediated virulence can be affected by HA glycosylation. Virulence in mice conferred by a short stalk NA was most evident when the HA had no glycosylation [38]. Although virulence in vivo is much more complicated than LTGF-β activation, studies are underway to examine the role of HA in LTGF-β. We hypothesize that although HA will not be directly involved in activation, it may influence the ability of NA to activate.

The question remains whether NA-mediated activation has an important biological role in TGF-β activation during influenza infection *in vivo*. At this time we can't definitively answer that question. What is intriguing is that the NA may influence the total levels of lung TGF-β during infection. Mice infected with HK/483 had a dramatic decrease in total LTGF-β levels by 2 dpi (from ∼2000 pg/ml to ∼1000 pg/ml). This phenotype was reversed with the HK/483 virus expressing the HK/486 NA. A similar trend was seen when HK/483 NA was expressed on HK/486; a significant decrease in total TGF-β levels. Studies are on-going to determine if this is due to a change in the cells in the lung associated with TGF-β secretion or if the viruses differentially regulate the known physiologic activators.

There are numerous physiologic TGF-β activators in the lung: the infected epithelium could release thrombospondin-1, proteases and matrix metalloproteases, or even reactive oxygen species (reviewed in [15], [21], [39]). Virus-induced injury to the epithelium can directly activate LTGF-β through the induction of apoptosis [40] or the upregulation of integrins [41]–[44], and immune cells have high levels of active TGF-β [45], [46]. Proteases may play a role in influenza virus-induced LTGF-β activation, especially during influenza infection *in vivo*, wherein cellular proteases are essential for influenza virus replication (reviewed in [47]–[50]). Proteases can be contaminants of viral preparations or even components of the viral membrane [29], [30]. Thus, only protease-free reagents were used in our assays, and a broad-spectrum PI cocktail partially blocked influenza virus and NA-mediated LTGF-β activation. Further studies confirmed that the broad-spectrum PI cocktail had no effect on either sialidase activity (as measured in the MUNANA assay) or directly on TGF-β detection in either assay (data not shown). However, we have not been able to identify the specific class of proteases or a potential cleavage site within LTGF-β by mass spectrometry (data not shown).

Since our initial attempts to construct H5 viruses that can activate TGF-β *in vivo* were unsuccessful, we evaluated the role of TGF-β in influenza pathogenesis by using a neutralizing antibody and administering exogenous TGF-β via an adenovirus vector. Mice administered TGF-β neutralizing antibody during HK/486 H5N1 infection had higher morbidity and mortality than mice treated with control virus or isotype IgG. This finding was not specific to H5N1 influenza viruses; inhibiting TGF-β activity during 2009 H1N1 infection also increased morbidity. Although mice exhibited clinical signs of illness, administration of exogenous TGF-β to HK/483-infected mice once at 24 hpi delayed morbidity and mortality. Administration of exogenous TGF-β 48 h pre-infection did not affect survival, and TGF-β–treated infected and non-infected mice had increased weight loss by 4 dpi, suggesting that the timing of TGF-β activation may be important.

Although we are still investigating the specific protective role of TGF-β during influenza infection, we did find that mice administered exogenous TGF-β had significantly lower titers within 2 dpi than untreated infected and AdDL70-treated mice. This decrease in viral load may contribute to the delayed morbidity observed, but was not sufficient to protect mice from severe infection. In contrast, depleting TGF-β during HK/486 infection had little to no significant effect on viral load until 8 dpi. Thus, mechanisms other than reduction of viral load may be involved in TGF-β–mediated modulation of influenza pathogenesis.

TGF-β serves as a global regulator of immunity by controlling the initiation and resolution of inflammatory responses (reviewed in [39], [46]). Thus, a pathogen that can regulate TGF-β activation could promote an immune-privileged state for itself within its host, as has been seen in the case of multiple parasitic, bacterial, and fungal pathogens (reviewed in [46], [51]–[55]). We postulate that failure of certain H5N1 influenza viruses to activate TGF-β [56], [57] may result in improper immune stimulation and resolution, contributing to exacerbated immunopathology for the host. Further investigation into specific immune cell activities and cytokine profiles during TGF-β modulation is required to fully elucidate the mechanisms of TGF-β regulation of influenza virus replication.

## Materials and Methods

### Ethics statement

All procedures involving animals were approved by the Southeast Poultry Research Laboratory (USDA-ARS), University of Wisconsin-Madison School of Medicine and Public Health, and the St. Jude Children's Research Hospital IACUCs and were in compliance with the Guide for the Care and Use of Laboratory Animals. These guidelines were established by the Institute of Laboratory Animal Resources and approved by the Governing Board of the U.S. National Research Council.

### Laboratory facility

All experiments in which H5N1 viruses were used were conducted in a Biosafety level 3 enhanced containment laboratory [58]. Investigators were required to wear appropriate respirator equipment (RACAL, Health and Safety Inc., Frederick, MD). Mice were housed in HEPA-filtered, negative pressure, vented isolation containers (M.I.C.E. ®, Animal Care Systems, Littleton, CO).

### Cell and virus propagation

A/Turkey/Wisconsin/68 (Tk/WI, H5N9), A/Gray Teal/Australia/2/79 (N4, H4N4), A/Swine/Nebraska/2/92 (Sw/NB, H1N1), A/Turkey/England/69 (Tk/Eng, H3N2), A/Turkey/Oregon/71 (Tk/Oreg, H7N3), A/Turkey/Ontario/6528/67 (Tk/Ont, H5N9), A/Mallard/Wisconsin/8/76 (Mal/WI, H1N1), A/Teal/Hong Kong/W312/97(Teal/HK, H6N1), and the 2009 H1N1 A/California/04/09 (CA/09) and A/Wisconsin/054/09 viruses were propagated in the allantoic cavity of 10-day-old specific pathogen-free embryonated chicken eggs (Sunnyside Farms, Beaver Dam, WI) at 37°C. Allantoic fluid was harvested, clarified by centrifugation and stored at −70°C. A/Puerto Rico/8 (PR8, H1N1), A/WSN/33 (WSN, H1N1), A/New Caledonia/20/99 (New Cal, H1N1), A/Hawaii/10/2002 (Hawaii, H1N2), A/Wyoming/3/2003 (Wyom, H3N2), A/Korea/770/2002 (Korea, H3N2), A/Aichi/2/68 (Aichi, H3N2), and the H5N1 viruses A/Hong Kong/156/97 (HK/156), A/Hong Kong/486/1997 (HK/486), A/Hong Kong/483/1997 (HK/483), A/Vietnam/1203/2004 (VN/1203), and A/Vietnam/1194/2004 (VN/1194) were propagated in Madin-Darby canine kidney (MDCK) cells as described previously [59]. Culture supernatants were harvested, clarified by centrifugation, and stored at −70°C. All viral titers were determined by 50% tissue culture infective dose (TCID_50_) analysis in MDCK cells and evaluated by the method of Reed and Muench [60]. MDCK cells were cultured in Eagle's minimal essential medium (MEM) supplemented with 2 mM glutamine (Mediatech, Manassas, VA), and 10% fetal bovine serum (FBS, Gemini Bio-Products, West Sacramento, CA). Mink lung epithelial cells stably transfected with the TGF-β-sensitive plasminogen activator inhibitor reporter construct (Mv1Lu-PAI cells, generous gift of Dr. Daniel Rifkin, New York University) were propagated in Dulbecco's modified Eagle's medium (DMEM) supplemented with 2 mM glutamine, 7% FBS, and 400 µg/ml Geneticin (G418, Calbiochem, La Jolla, CA).

### Purification of influenza virus and NA

Gray Teal influenza virus was purified by sucrose gradient ultracentrifugation [61]. Purified Gray Teal NA was obtained through the NIH Biodefense and Emerging Infections Research Resources Repository, NIAID, NIH: N4 Neuraminidase (NA) Protein from Influenza Virus, A/grey teal/Australia/2/79 (H4N4), Recombinant from baculovirus, NR-656 (BEI Resources, Manassas, VA). Briefly, it was expressed in Sf9 cells using a baculovirus expression vector system and purified using conventional chromatographic techniques.

### NA activity assay

NA enzymatic activity was determined by the MUNANA (2-(4-methylumbelliferyl)-α-d-*N*-acetylneuraminic acid) assay as described previously [62]. NA inhibition was assayed with purified NA, and virus was standardized to equivalent NA enzyme activity and incubated for 1 h at 37°C with oseltamivir carboxylate (0–1000 nM, generous gift of Hoffman La-Roche, Inc., Nutley, NJ).

### TGF-β assays

TGF-β activity was assessed by the plasminogen activator inhibitor-luciferase (PAI/L) bioassay or by a TGF-β-specific ELISA following manufacturer's instructions (R&D Systems, Minneapolis, MN). The ELISA is a quantitative sandwich immunoassay where a monoclonal antibody specific for the active region of TGF-β1 is coated onto a microplate and any bound TGF-β1 is detected with an enzyme-linked polyclonal antibody specific for TGF-β1. It will not detect the latent form of TGF-β1. The PAI/L bioassay was performed as previously described [63], with several modifications. Briefly, 2×10^4^ Mv1Lu-PAI cells per well of 96-well plates were incubated overnight, washed with PBS, and incubated with 100 µl/well test sample for 5 h at 37°C, 5% CO_2_. After washing, cells were lysed and luciferase activity measured by using a luciferase assay substrate (Promega, Madison, WI) on a Turner Biosystems 20/20^n^ luminometer (Turner Biosystems Instruments, Sunnyvale, CA). Test samples included 10 ng/ml recombinant LTGF-β1 (rLTGF-β1, R&D Systems) incubated with different concentrations of low-protease-content *Clostridium perfringens-*purified NA (Roche Applied Sciences, Indianapolis, IN), purified NA, or influenza virus in serum-free DMEM containing 0.1% BSA for 1 h at 37°C. To generate TGF-β1 standard curves, 2-fold dilutions of active TGF-β1 (0–1000 pg/ml, R&D Systems) in DMEM containing 0.1% BSA were added to Mv1Lu-PAI cells. To determine the role of NA activity or proteases, rLTGF-β1 was pre-incubated for 1 h at 37°C with different NA activities of bNA, purified influenza virus, or viral NA (as noted in figures and figure legends) in the presence of oseltamivir carboxylate (10 nM) or 1× EDTA-free PI cocktail (Pierce, Rockford, IL).

### Protease assays

To examine the role of individual proteases, 10^6^ TCID_50_ units/ml of purified Tk/WI influenza virus was pre-incubated for 1 h at 37°C with bestatin (20–1000 nM; Sigma), leupeptin (10–100 µM; Sigma), or GM 1489 (1–500 nM; Calbiochem), followed by incubation with 10 ng/ml rLTGF-β1 for 1 h at 37°C. LTGF-β1 activation was determined by the PAI/L assay. All protease inhibitors were used within their effective inhibitory concentrations, as determined by the manufacturer. To test for the presence of proteases in experimental reagents, including purified virus, proteins, and inhibitors, a thiocarbamoyl-casein derivative-based assay was used as per manufacturer's instructions (Calbiochem) in the presence or absence of 1× protease inhibitor cocktail (Pierce).

### LTGF-β Western blot and sialic acid content

rLTGF-β (0.4 µg) or rLAP (0.5 µg, R&D Systems) was incubated with PBS, HCl (final pH of 2), purified Gray Teal virus (2 µg), purified Gray Teal NA (0.5 µg), or bNA either alone or pre-incubated with NAi (1 µM) or 1× PI for 1 h at 37°C as described previously. Samples were then separated on a 5%–20% SDS-PAGE gel under reducing conditions. After transferring to nitrocellulose, blots were blocked in 2% non-fat dry milk in Tris-buffered saline plus 1% Tween 20 (TTBS) for 1 h at room temperature and probed for LAP with mouse-anti-LAP (1∶500, R&D Systems) in TTBS for 1 h at room temperature. Blots were washed and incubated with goat anti-mouse-HRP (1∶5000, Jackson Laboratories, Bar Harbor, ME). To examine the sialic acids present on LAP, TGF-β samples were prepared as described above and blots were probed with DIG-labeled MAA (1∶200), which recognizes α2,3-linked sialic acids, or SNA (1∶1000), which recognizes α2,6-linked sialic acids, for 1 h at room temperature (DIG glycan differentiation kit, Roche Applied Science). Lectins were visualized by staining with anti-DIG-AP. After detection, blots were analyzed by Western blotting for LAP, as described above.

### Reverse genetics

H5N1 reverse genetic viruses were generated by the RNA polymerase I reverse genetics system [64]. The PR8 and CA/09 virus expressing HK/483 NA was constructed by using the eight-plasmid system as previously described [65]. P1 viral stocks were generated, the *NA* genes sequenced to ensure that no spurious mutations arose during viral propagation, stiters determined by TCID_50_ analysis in MDCK cells, and NA activity quantitated by the MUNANA assay as described above. To determine TGF-β levels with the reassortant viruses, BALB/c mice were lightly anesthetized and inoculated with 10^4^ TCID_50_ units of the different reassortant viruses or PBS alone, as described below.

### TGF-β modulation *in vivo*

To deplete TGF-β activity during HK/486 and CA/09 virus infection, 4- to 6-week-old BALB/c mice (Charles River Laboratories, Wilmington, MA) were intraperitoneally (i.p.) inoculated with PBS, anti-TGF-β neutralizing antibody 1D11, or isotype-matched mouse IgG (0.5 mg per mouse, Sigma) in PBS 6 to 48 h pre-infection and then every 48 hpi. Mice were then intranasally (i.n.) inoculated with 25 µl PBS or 10^5^ TCID_50_ virus. 1D11 was either purchased (R&D Systems) or purified from the 1D11 hybridoma (ATCC# 1D11.16.8). 1D11 produces IgG1 antibodies that neutralize all 3 mammalian TGF-β isoforms (β1, β2, β3) [66]. IgG was purified from cell culture supernatants by T-Gel (Pierce), potential endotoxin contaminations removed by Detoxi Gel Endotoxin Removing Gel (Pierce), and purified IgG concentrated and buffer exchanged with Amicon Ultra-15 concentrators (Millipore, Bedford, MA). The endotoxin levels were less than 0.2 EU/mg as measured by the Biowhittaker QCL-1000 assay (Biowhittaker, Walkersville, MD).

Exogenous active TGF-β1 was administered to mice by infection with a replication-defective adenovirus expressing active TGF-β1 (AdTGFβ^223/225^) or the control vector (AdDL70) as described previously [67], [68]. Briefly, full-length porcine TGFββ1 cDNA (differing from murine TGF-β1 by 1 amino acid) was mutated at serine 223 and 225 (TGF-β^223/225^) to render the protein constitutively active and expressed in a recombinant, replication-deficient type-5 adenovirus. The replication-deficient virus (AdTGFβ^223/225^) was purified by cesium chloride (CsCl) gradient centrifugation and concentrated by using a Sephadex PB-10 chromatography column. Mice were i.n. inoculated with 10^8^ PFUs of active AdTGFβ^223/225^ or AdDL70 either 48 h pre- or 24 h post-infection with 10^4^ TCID_50_ units of HK/483 virus. Mice were monitored daily and weighed every 48 h post-infection. At different time points post-infection, 2 mice from the control group and 3 mice from the experimental group were euthanized and lungs collected. Tissues were homogenized in cold PBS, and clarified tissue homogenates were tested for TGF-β levels, using a mouse-specific ELISA (R&D Systems) or viral titers by TCID_50_ analysis on MDCK cells.

### Statistical analyses

Statistical significance of data was determined by using analysis of variance (ANOVA) or Student's *t*- test on GraphPad Prism (San Diego, CA). All assays were run in triplicate and are representative of at least 2 separate experiments. Error bars represent standard deviation, and statistical significance was defined as a *p* value of less than 0.05.

## Supporting Information

Figure S1 Gray Teal NA does not cleave α2-6 linked sialic acids. rLTGF-β (0.4 µg) was incubated with PBS, bNA, or purified GT NA (0.5 µg) for 1 h at 37°C. Proteins were separated by SDS-PAGE under reducing conditions, transferred to nitrocellulose, and probed with digoxigenin-labeled SNA lectin. bNA and GT NA alone were run as controls.
(0.54 MB EPS)

Figure S2 Endogenous protease activity in influenza virus stocks. FTC-casein derivative was incubated with PBS, trypsin (positive control), bNA (30,000 RFU), or 90,000 RFU of different influenza virus strains or GT NA in the presence (red bar) or absence (black bar) of 1× PI cocktail for 24 h at 37°C. Fluorescence measured at excitation 490 nm and emission 525 nm. Results are expressed as fold increase over the negative control. Asterisk (*) indicates significant increase as compared with the negative control.
(0.44 MB EPS)

Figure S3 Pretreatment with exogenous TGF-β does not prolong survival in HK/483-infected mice. BALB/c mice (4–6 weeks) were inoculated i.n. with PBS (control) or 10^8^ PFU/mouse of a TGF-β-expressing adenovirus (AdTGFβ^223/225^) or an adenovirus vector control (AdDL70) (*n* = 12) 48 h before inoculation with 10^4^ TCID_50_ units of HK/483 virus (*n* = 12). Mice inoculated with AdDL70 or AdTGFβ^223/225^ alone served as controls (*n* = 10). Weights (A) and survival (B) were monitored for 10 dpi. Asterisk (*) indicates significant difference in weight loss as compared to HK/483 infected mice.
(0.57 MB EPS)

## References

[1] Morty RE, Konigshoff M, Eickelberg O (2009). Transforming Growth Factor-{beta} Signaling across Ages: From Distorted Lung Development to Chronic Obstructive Pulmonary Disease.. Proc Am Thorac Soc.

[2] Taipale J, Miyazono K, Heldin CH, Keski-Oja J (1994). Latent transforming growth factor-beta 1 associates to fibroblast extracellular matrix via latent TGF-beta binding protein.. J Cell Biol.

[3] Piek E, Heldin CH, Ten Dijke P (1999). Specificity, diversity, and regulation in TGF-beta superfamily signaling.. Faseb J.

[4] Gentry LE, Lioubin MN, Purchio AF, Marquardt H (1988). Molecular events in the processing of recombinant type 1 pre-pro- transforming growth factor beta to the mature polypeptide.. Mol Cell Biol.

[5] Lawrence DA, Pircher R, Kryceve-Martinerie C, Jullien P (1984). Normal embryo fibroblasts release transforming growth factors in a latent form.. J Cell Physiol.

[6] Munger JS, Harpel JG, Gleizes PE, Mazzieri R, Nunes I (1997). Latent transforming growth factor-beta: structural features and mechanisms of activation.. Kidney Int.

[7] Sha X, Yang L, Gentry LE (1991). Identification and analysis of discrete functional domains in the pro region of pre-pro-transforming growth factor beta 1.. J Cell Biol.

[8] Young GD, Murphy-Ullrich JE (2004). Molecular Interactions That Confer Latency to Transforming Growth Factor-{beta}.. J Biol Chem.

[9] Annes JP, Munger JS, Rifkin DB (2003). Making sense of latent TGFbeta activation.. J Cell Sci.

[10] Aluwihare P, Munger JS (2008). What the lung has taught us about latent TGF-beta activation.. Am J Respir Cell Mol Biol.

[11] Barcellos-Hoff MH, Dix TA (1996). Redox-mediated activation of latent transforming growth factor-beta 1.. Mol Endocrinol.

[12] Vodovotz Y, Chesler L, Chong H, Kim SJ, Simpson JT (1999). Regulation of transforming growth factor beta1 by nitric oxide.. Cancer Res.

[13] Brown PD, Wakefield LM, Levinson AD, Sporn MB (1990). Physicochemical activation of recombinant latent transforming growth factor-beta's 1, 2, and 3.. Growth Factors.

[14] Lawrence DA, Pircher R, Jullien P (1985). Conversion of a high molecular weight latent beta-TGF from chicken embryo fibroblasts into a low molecular weight active beta-TGF under acidic conditions.. Biochem Biophys Res Commun.

[15] Jenkins G (2008). The role of proteases in transforming growth factor-beta activation.. Int J Biochem Cell Biol.

[16] Schultz-Cherry S, Chen H, Mosher DF, Misenheimer TM, Krutzsch HC (1995). Regulation of transforming growth factor-beta activation by discrete sequences of thrombospondin 1.. J Biol Chem.

[17] Schultz-Cherry S, Lawler J, Murphy-Ullrich JE (1994). The type 1 repeats of thrombospondin 1 activate latent transforming growth factor-beta.. J Biol Chem.

[18] Schultz-Cherry S, Ribeiro S, Gentry L, Murphy-Ullrich JE (1994). Thrombospondin binds and activates the small and large forms of latent transforming growth factor-beta in a chemically defined system.. J Biol Chem.

[19] Schultz-Cherry S, Murphy-Ullrich JE (1993). Thrombospondin causes activation of latent transforming growth factor- beta secreted by endothelial cells by a novel mechanism [published erratum appears in J Cell Biol 1993 Sep;122(5):following 1143].. J Cell Biol.

[20] Murphy-Ullrich JE, Poczatek M (2000). Activation of latent TGF-beta by thrombospondin-1: mechanisms and physiology.. Cytokine Growth Factor Rev.

[21] Taylor AW (2009). Review of the activation of TGF-{beta} in immunity.. J Leukoc Biol.

[22] Koth LL, Alex B, Hawgood S, Nead MA, Sheppard D (2007). Integrin beta6 mediates phospholipid and collectin homeostasis by activation of latent TGF-beta1.. Am J Respir Cell Mol Biol.

[23] Gantt KR, Schultz-Cherry S, Rodriguez N, Jeronimo SM, Nascimento ET (2003). Activation of TGF-beta by Leishmania chagasi: importance for parasite survival in macrophages.. J Immunol.

[24] Omer FM, de Souza JB, Corran PH, Sultan AA, Riley EM (2003). Activation of transforming growth factor beta by malaria parasite-derived metalloproteinases and a thrombospondin-like molecule.. J Exp Med.

[25] Aung H, Wu M, Johnson JL, Hirsch CS, Toossi Z (2005). Bioactivation of latent transforming growth factor beta1 by Mycobacterium tuberculosis in human mononuclear phagocytes.. Scand J Immunol.

[26] Waghabi MC, Keramidas M, Feige JJ, Araujo-Jorge TC, Bailly S (2005). Activation of transforming growth factor beta by Trypanosoma cruzi.. Cell Microbiol.

[27] Schultz-Cherry S, Hinshaw VS (1996). Influenza virus neuraminidase activates latent transforming growth factor beta.. J Virol.

[28] Kim NY, Kim HG, Kim YH, Chung IS, Yang JM (2008). Expression and characterization of human N-acetylglucosaminyltransferases and alpha2,3-sialyltransferase in insect cells for in vitro glycosylation of recombinant erythropoietin.. J Microbiol Biotechnol.

[29] Benureau Y, Huet JC, Charpilienne A, Poncet D, Cohen J (2005). Trypsin is associated with the rotavirus capsid and is activated by solubilization of outer capsid proteins.. J Gen Virol.

[30] Shaw ML, Stone KL, Colangelo CM, Gulcicek EE, Palese P (2008). Cellular proteins in influenza virus particles.. PLoS Pathog.

[31] Hoffmann E, Stech J, Leneva I, Krauss S, Scholtissek C (2000). Characterization of the influenza A virus gene pool in avian species in southern China: was H6N1 a derivative or a precursor of H5N1?. J Virol.

[32] Dybing JK, Schultz-Cherry S, Swayne DE, Suarez DL, Perdue ML (2000). Distinct pathogenesis of hong kong-origin H5N1 viruses in mice compared to that of other highly pathogenic H5 avian influenza viruses.. J Virol.

[33] Taylor G (1996). Sialidases: structures, biological significance and therapeutic potential.. Current Opinion in Structural Biology.

[34] Miyazono K, Heldin CH (1989). Role for carbohydrate structures in TGF-beta 1 latency.. Nature.

[35] Wakefield LM, Winokur TS, Hollands RS, Christopherson K, Levinson AD (1990). Recombinant latent transforming growth factor beta 1 has a longer plasma half-life in rats than active transforming growth factor beta 1, and a different tissue distribution.. J Clin Invest.

[36] Zou Z, Sun PD (2006). An improved recombinant mammalian cell expression system for human transforming growth factor-[beta]2 and -[beta]3 preparations.. Protein Expression and Purification.

[37] Cauthen AN, Swayne DE, Schultz-Cherry S, Perdue ML, Suarez DL (2000). Continued circulation in China of highly pathogenic avian influenza viruses encoding the hemagglutinin gene associated with the 1997 H5N1 outbreak in poultry and humans.. J Virol.

[38] Matsuoka Y, Swayne DE, Thomas C, Rameix-Welti MA, Naffakh N (2009). Neuraminidase stalk length and additional glycosylation of the hemagglutinin influence the virulence of influenza H5N1 viruses for mice.. J Virol.

[39] Koli K, Myllarniemi M, Keski-Oja J, Kinnula VL (2008). Transforming growth factor-beta activation in the lung: focus on fibrosis and reactive oxygen species.. Antioxid Redox Signal.

[40] Victor T, Solovyan JK-O (2006). Proteolytic activation of latent TGF-? precedes caspase-3 activation and enhances apoptotic death of lung epithelial cells.. J Cell Phys.

[41] Munger JS, Huang X, Kawakatsu H, Griffiths MJ, Dalton SL (1999). The integrin alpha v beta 6 binds and activates latent TGF beta 1: a mechanism for regulating pulmonary inflammation and fibrosis.. Cell.

[42] Pittet JF, Griffiths MJ, Geiser T, Kaminski N, Dalton SL (2001). TGF-beta is a critical mediator of acute lung injury.. J Clin Invest.

[43] Sheppard D (2001). Integrin-mediated activation of transforming growth factor-beta(1) in pulmonary fibrosis.. Chest.

[44] Sheppard D (2005). Integrin-mediated activation of latent transforming growth factor beta.. Cancer Metastasis Rev.

[45] Bosse Y, Rola-Pleszczynski M (2007). Controversy surrounding the increased expression of TGF beta 1 in asthma.. Respir Res.

[46] Li MO, Wan YY, Sanjabi S, Robertson AK, Flavell RA (2006). Transforming growth factor-beta regulation of immune responses.. Annu Rev Immunol.

[47] Kido H, Okumura Y, Yamada H, Le TQ, Yano M (2007). Proteases essential for human influenza virus entry into cells and their inhibitors as potential therapeutic agents.. Curr Pharm Des.

[48] Zhang L, Katz JM, Gwinn M, Dowling NF, Khoury MJ (2009). Systems-based candidate genes for human response to influenza infection.. Infection, Genetics and Evolution.

[49] Kido H, Murakami M, Oba K, Chen Y, Towatari T (1999). Cellular proteinases trigger the infectivity of the influenza A and Sendai viruses.. Mol Cells.

[50] Rowe RK, Brody SL, Pekosz A (2004). Differentiated cultures of primary hamster tracheal airway epithelial cells.. In Vitro Cell Dev Biol Anim.

[51] Gordon KJ, Blobe GC (2008). Role of transforming growth factor-beta superfamily signaling pathways in human disease.. Biochim Biophys Acta.

[52] Fitzpatrick DR, Bielefeldt-Ohmann H (1999). Transforming growth factor beta in infectious disease: always there for the host and the pathogen.. Trends Microbiol.

[53] Reed SG (1999). TGF-beta in infections and infectious diseases.. Microbes Infect.

[54] Omer FM, Kurtzhals JA, Riley EM (2000). Maintaining the immunological balance in parasitic infections: a role for TGF-beta?. Parasitol Today.

[55] Omer FM, Riley EM (1998). Transforming growth factor beta production is inversely correlated with severity of murine malaria infection.. J Exp Med.

[56] Baigent SJ, McCauley JW (2001). Glycosylation of haemagglutinin and stalk-length of neuraminidase combine to regulate the growth of avian influenza viruses in tissue culture.. Virus Res.

[57] Shtyrya Y, Mochalova L, Voznova G, Rudneva I, Shilov A (2009). Adjustment of receptor-binding and neuraminidase substrate specificities in avian-human reassortant influenza viruses.. Glycoconj J.

[58] Richmond JY, McKinney RW (1993). Biosafety in microbiological and biomedical laboratories.

[59] Jones JC, Turpin EA, Bultmann H, Brandt CR, Schultz-Cherry S (2006). Inhibition of Influenza Virus Infection by a Novel Antiviral Peptide That Targets Viral Attachment to Cells.. J Virol.

[60] Reed LJ, Muench H (1938). A simple method of estimating fifty percent endpoints.. Am J Hyg.

[61] Johansson BE, Bucher DJ, Kilbourne ED (1989). Purified influenza virus hemagglutinin and neuraminidase are equivalent in stimulation of antibody response but induce contrasting types of immunity to infection.. J Virol.

[62] Potier M, Mameli L, Belisle M, Dallaire L, Melancon SB (1979). Fluorometric assay of neuraminidase with a sodium (4-methylumbelliferyl-alpha-D-N-acetylneuraminate) substrate.. Analytical Biochemistry.

[63] Abe M, Harpel JG, Metz CN, Nunes I, Loskutoff DJ (1994). An assay for transforming growth factor-beta using cells transfected with a plasminogen activator inhibitor-1 promoter-luciferase construct.. Anal Biochem.

[64] Neumann G, Watanabe T, Ito H, Watanabe S, Goto H (1999). Generation of influenza A viruses entirely from cloned cDNAs.. Proc Natl Acad Sci U S A.

[65] Hoffmann E, Krauss S, Perez D, Webby R, Webster RG (2002). Eight-plasmid system for rapid generation of influenza virus vaccines.. Vaccine.

[66] Dasch J, Pace D, Waegell W, Inenaga D, Ellingsworth L (1989). Monoclonal antibodies recognizing transforming growth factor-beta. Bioactivity neutralization and transforming growth factor beta 2 affinity purification.. J Immunol.

[67] Gauldie J, Galt T, Bonniaud P, Robbins C, Kelly M (2003). Transfer of the active form of transforming growth factor-beta 1 gene to newborn rat lung induces changes consistent with bronchopulmonary dysplasia.. Am J Pathol.

[68] Robertson JV, Nathu Z, Najjar A, Dwivedi D, Gauldie J (2007). Adenoviral gene transfer of bioactive TGFbeta1 to the rodent eye as a novel model for anterior subcapsular cataract.. Mol Vis.

